# Employee-Driven Innovation as an Approach to Health System Strengthening in LMICs: Comment on "Employee-Driven Innovation in Health Organizations: Insights From a Scoping Review"

**DOI:** 10.34172/ijhpm.8637

**Published:** 2024-12-14

**Authors:** Lindi van Niekerk

**Affiliations:** Chembe Collaborative, Los Angeles, CA, USA.

**Keywords:** Human-Resources, Bottom-up Innovation, Organizational Culture, Health Systems

## Abstract

Cadeddu and colleagues’ scoping review, *Employee-Driven Innovation in Health Organizations*, provides a valuable reframing of healthcare workers’ roles beyond service delivery and positions them as key contributors to organizational and systems innovation. Key gaps identified in the literature through their scoping review include limited evidence from low- and middle-income countries (LMICs) and an incomplete understanding of how top-down or hybrid employee-driven innovation (EDI) processes can effectively enable bottom-up innovation. This commentary provides possible reasons for the limited published evidence from LMICs and, uses the framework by Caddedu et al, to address the knowledge gaps by presenting examples of EDI processes from LMICs, as well as discussing the barriers and enablers of EDI in these settings. Examples from LMICs demonstrate that EDI not only drives solutions to enhance the efficiency and quality of care but also plays a pivotal role in fostering positive organizational cultures within health systems.

 Cadeddu and colleagues’ scoping review titled “Employee-Driven Innovation in Health Organizations” offers a much-needed contribution as it reframes the role of healthcare workers beyond the delivery of services and highlights their role as potential contributors to health system strengthening through innovation.^1^ The authors conceptualize employee-driven innovation (EDI) as a participatory, learning process that leverages the contributions of “ordinary” employees (clinical, non-clinical, or administrative) to achieve innovation outcomes. The paper provides a synthesized framework capturing the processes, the macro-level, organizational, and individual enablers and barriers, as well as potential benefits/outcomes of EDI.

 The authors noted several gaps in the current literature, particularly the limited evidence from low- and middle-income countries (LMICs), with only three studies from LMICs versus 49 from high-income countries. This may stem from omitting search terms like “social innovation,” “operational innovation,” and “intrapreneurship.” Additionally, many LMIC frontline workers view their efforts to improve care simply as routine problem-solving rather than innovation, often occurring under the radar of management. Significant demands of LMIC workers, leave little time or resources to document or publish innovative initiatives.

 Furthermore, the authors emphasize the importance of and limited current knowledge on how top-down processes can support hybrid or bottom-up EDI, as collaboration between management and employees is essential for sustainable, scalable innovation across the health system. Building on the authors’ well-synthesized framework of enablers and barriers, this commentary aims to address knowledge gaps by sharing documented EDI examples from selected LMICs: South Africa, Malawi, Cameroon, Pakistan, and regionally across countries in Latin America. Evidence from these examples was missed in the initial scoping review.

 In the above-mentioned settings, innovation was required to address the access gap to health services, the suboptimal quality of health services, and the social determinants responsible for ill health in low-income, uninsured, or marginalized patient populations, especially given limited financial resources for health, acute health worker shortages, low socio-economic conditions, and high disease burdens.^2-6^ Top-down (management or external initiative) and hybrid (employee-initiated, management-formalized) EDI processes were adopted to stimulate bottom-up innovation.^1^ Methodologies drawn on included human-centered design, social innovation, positive organizational development, and learning via research. Each setting’s EDI approach is outlined below, followed by an analysis of enablers and barriers at individual, organizational, and macro levels, concluding with insights on EDI’s potential benefits for LMICs.

 At an 800-bed public hospital in Cape Town, South Africa, the first hospital-based innovation program adopted a hybrid EDI process (2014-2015) through a collaboration involving the hospital’s executive management, charity board, and two university faculties.^7^ Designed to improve care delivery and employee morale, the program used ethnographic methods to identify eight critical care challenges faced by staff and common across departments.^7^ Staff teams received an opportunity to propose innovative ideas to address these challenges, generating 25 staff-led innovation proposals.^8^ After a selection process, ten cross-disciplinary teams were supported for nine months to develop practical, measurable projects, and in addition, 13 existing staff innovations were recognized and celebrated.^9,10^

 As part of a global initiative to transform hospital care through innovation (2022–ongoing), hospitals in Cameroon and Pakistan implemented top-down EDI processes to support collaborative frontline-driven solutions. Recognizing the pandemic’s impact on healthcare workers and gaps in hospital-community integration, an international multilateral health agency partnered with the representative Ministry of Health and assembled cross-disciplinary frontline teams in nine participating hospitals. Teams received training in innovation approaches and tools, and each hospital team designed an innovative solution in response to their local care delivery challenge.

 Since 2015, coordinated efforts have been made across the Latin America and Caribbean region to build a local social innovation ecosystem, through a targeted initiative led by a regional multilateral organization in collaboration with two university research centers in Colombia and Honduras.^11-13^ Using a hybrid EDI process, this initiative recognized and supported existing health-related social innovations and conducted five regional crowdsourcing calls to identify innovative health solutions from diverse organizations, including nonprofits, government health departments, universities, and community groups.^14^ Crowdsourcing was found to be an effective strategy to identify community-level unpublished innovations. Recognition, alongside capacity-building workshops from university partners, helped innovators secure funding, gain government support, scale their initiatives, and develop sustainable evaluation practices. A notable example identified was an integrated primary care program in Sumapaz, on the rural outskirts of Bogotá, Colombia, where public health and local primary care staff collaborated to address the rural community’s health, environmental, and nutritional needs through co-learning and community involvement.^15^

 A final example of a hybrid innovation process is the Malawi Ministry of Health’s initiative to address care access challenges by soliciting citizen ideas and partnering with an international non-governmental organization and telecom company to pilot, evaluate, and scale a selected innovation.^16^ The resulting solution—a national, nurse-led telephonic health advice service accessible to all Malawians at no cost— became integral to the government’s COVID-19 response. This initiative highlights the potential of citizen-driven innovation, supported by non-state organizations and government, as an effective approach to health systems strengthening.^17^

## Employee-Driven Innovation Enablers in LMICs

 Several cross-cutting enablers were critical in the above-described LMIC EDI examples. At an individual level, Cadeddu et al refer to a proactive employee personality and work context.^1^ Research conducted on the EDI process in Malawi studied the individuals involved and found them to have more than just a proactive or charismatic personality. They operated as institutional entrepreneurs, characterized as “actors who initiate divergent changes in the institutional context and who actively participate in the implementation.”^18^ Despite being in challenging work contexts, these institutional entrepreneurs were future-oriented visionary individuals with a high-hope quotient, internal agency, and a high level of humility. These characteristics enabled them to effectively engage their social networks and social capital to realize innovative ideas.^17^

 Contrary to the findings by Cadeddu et al, the most important organizational enabler of EDI was not the availability of resources, rather the staff’s direct manager permitting them to innovate. Staff had many ideas but often lacked the courage to implement their ideas, and felt the need to engage in innovation in secret as they did not believe it was in their remit. A second LMIC organizational enabler was recognition coupled with celebration. LMIC EDI processes were frequently pursued with minimal or no financial resources, with management’s recognition being a sufficient incentive. In Latin America, the formal recognition gave innovators motivation to pursue their innovative initiatives despite the challenges and roadblocks experienced.^11^ Once permission and recognition were present, providing technical and educational support became the next important enabler. Staff were eager to learn and understand how to turn problems into measurable solutions. In the above-mentioned examples, initial innovation training was done in person, but post-pandemic, creative innovation toolkits with accompanying educational videos and podcasts focusing on different innovation skills and journeys.^19^ A fourth organizational-level EDI enabler in LMICs was the establishment of innovation project partnerships between staff and patients, families, community organizations, and the private sector. These partnerships provide necessary material resources and valuable relational and motivational encouragement.

 At a macro-level, LMICs did not require the government to initiate or lead the EDI process, as found by Cadeddu et al, but the visible participation of the government in the EDI process served as an important enabler. This participation took various forms: rewarding/recognition, attending staff innovation presentations, working with staff innovators to develop their projects to be ‘fit for system’ from the start, and being willing to adopt and institutionalize impactful solutions as part of the national health system.^17^

## Employee-Driven Innovation Barriers in LMICs

 Barriers hindering EDI in LMIC health systems are plentiful. While similar barriers, such as those described by Cadeddu et al were present, such as a limited support system, a lack of incentives, minimal managerial support, and staff perceptions of additional work, the most prevailing EDI barrier are entrenched mindsets of senior leaders. Health systems in LMICs often operate within rigid hierarchies rooted in colonial legacies, where an individualistic, expert-driven, and efficiency-focused approach dominates, perpetuating power imbalances and disciplinary silos. Senior managers often and unintentionally undermine EDI by prescribing or dictating solutions instead of allowing their staff to experiment with their innovations. Culturally, staff at lower levels find it challenging to contradict or oppose a senior leader, and doing so could be considered disrespectful. Senior leaders do not often create safe spaces where the voices of lower staff cadres can be heard. EDI requires a shared leadership style that fosters mutual respect and appreciation as well as values collaborative decision-making and action.^20^

 A further challenge in LMICs, is the resistance of senior health leaders to fully integrate impactful initiatives as part of the national health system. Self-reflective government representatives have attributed this hesitation, not to a lack of interest in innovation, but to a need for practical support and guidance to effectively adapt and embed these initiatives. The Malawian EDI example demonstrates what is possible when senior leaders adopt a mindset characterized by humility, hope, and openness to learning from diverse health system stakeholders. The Malawian health system notably integrates national and indigenous principles, emphasizing collectivism and local ownership as essential to embedding innovation within the system.^21^

## EDI benefits in LMICs

 Cadeddu et al found that EDI programs improve efficiency, quality of care, and cost reduction. However, in LMICs, the primary benefit of EDI programs often lies in fostering a positive organizational culture within health organizations. This contrasts with traditional literature that views positive culture as a precursor to innovation. In all LMIC examples, EDI programs positively impacted relationship between management and staff, staff team dynamics, and self-perception. Staff members viewed leadership investment in EDI as an expression of support, as one Cameroonian health worker noted, “*It is so cool for me to think that the management of this institution believes in us to improve and is willing to give us a space, people and give this commitment to us.”* A South African health worker valued the change in interpersonal dynamics among staff, “*It (the innovation program) showed me the importance of relationships with my co-workers, in communication, teamwork, and coherence…we talk differently to each other now.” *A common shared experience of staff was a mindset shift that occurred for them, as well summarized by one Pakistani health worker: *“After learning about innovation, we realized that there are multiple solutions which did not need financing and it only requires a change of thinking. It motivated us; it changed our perception. The program helped me see a bigger picture, not just see myself as a medical doctor but do something bigger than just being in a consultation room.” *

 Despite evidence linking organizational culture with an improvement in patient outcomes,^22^ LMIC health organizations and systems have yet to invest in cultivating more collaborative, hope-filled, and agentic cultures marked by high-quality relationships between staff and between staff and management. Implementing hybrid and top-down EDI processes offers a pathway to fostering a positive organizational culture. EDI in LMICs is often seen as a costly luxury; however, significant enablers and barriers are achievable at little or no additional cost. Whether in LMICs, or high-income countries, the greatest prerequisite for EDI is a shift in perspective—seeing health staff not only as care providers but as system enhancers with the agency, capacity, and resources to contribute meaningfully.

## Acknowledgements

 Acknowledgments (listed alphabetically): I acknowledge partners who have collaborated in the implementation of the case examples described and those who provided inputs on this paper: Jackie Alger, Tania Bissouma-Ledjou, Carla Pamela Blauvelt, Emilie Calvello-Hynes, Amy Clarke, Luis Gabriel Cuervo, Maria-Isabel Echavarria, Nedson Fosiko, Ann-lise Guisset, Boris Kuomonge, Andrew Likaka, Patrice Ngangue, Don Mathanga, Idrisu Mbuih, Ulrich Meyer-Hollings, Barwani Msiska, Afifa Munawar, Bavna Patel, Peter J. Raubenheimer, Rosalie Thompson, and Claudi van Niekerk.

## Ethical issues

 Not applicable.

## Conflicts of interest

 Author declares that she has no conflicts of interest.
